# Technological Challenges and Future Issues for the Detection of Circulating MicroRNAs in Patients With Cancer

**DOI:** 10.3389/fchem.2019.00815

**Published:** 2019-11-28

**Authors:** Jean Cacheux, Aurélien Bancaud, Thierry Leichlé, Pierre Cordelier

**Affiliations:** ^1^LAAS-CNRS, Université de Toulouse, CNRS, Toulouse, France; ^2^Université Fédérale de Toulouse Midi-Pyrénées, Université Toulouse III Paul Sabatier, CRCT, Toulouse, France

**Keywords:** miRNA, sample preparation, micro- and nano-technology, biosensors, cancer

## Abstract

In the era of precision medicine, the success of clinical trials, notably for patients diagnosed with cancer, strongly relies on biomarkers with pristine clinical value but also on robust and versatile analytical technologies to ensure proper patients' stratification and treatment. In this review, we will first address whether plasmatic and salivary microRNAs can be considered as a reliable source of biomarkers for cancer diagnosis and prognosis. We will then discuss the pre-analytical steps preceding miRNA quantification (from isolation to purification), and how such process could be biased and time-consuming. Next, we will review the most recent tools derived from micro- and nano-technologies for microRNA detection available to date and how they may compete with current standards. This review will prioritize publications using relevant biological samples. The significance of various physical transduction schemes (mechanical, optical, electrical, etc.) for biological detection will be compared, and pros and cons of each method will be widely discussed. Finally, we will debate on how micro and nanotechnologies could widespread the use of biomarkers in modern medicine, to help manage patients with serious diseases such as cancer.

## Biogenesis and Function of microRNAs

MicroRNAs (miRNAs) are small RNA molecules that inhibit messenger RNA (mRNA) translation by binding to 3′-untranslated region (Bartel, 2004; Kim et al., 2009; Redis et al., 2012). These molecules are tightly involved in the regulation of many physiological processes including development, proliferation, invasion, and apoptosis. The first miRNA molecule, *Lin-4*, was discovered in 1993 in *Caenorhabditis elegans* (*C. elegans*) roundworms (Almeida et al., 2011). It was only seven years later, in 2000, that the second miRNA, *Let-7*, was identified in the same animal (Pasquinelli et al., 2000). In 2001, such “small RNAs” were coined as miRNAs by Ruvkun and coll (Ruvkun, 2001). Today, the existence of nearly thirty thousand miRNA sequences has been demonstrated in more than 200 eukaryotic species, some of them being conserved between species (miRBase).

MiRNAs are first encoded by RNA polymerase II from DNA as long RNA transcripts (primary RNA molecules, pri-miRNAs) of several thousand nucleotides (Cai et al., 2004). Several pathways give rise to mature miRNA (Miyoshi et al., 2010). One of the best documented pathway starts with the processing of pri-miRNAs into smaller sequences folded in hairpin structure ranging from 60 to 110 nucleotides (miRNA precursors, pre-miRNAs) by RNase III Drosha (Lee et al., 2003). These precursors are then exported from the cell nucleus to the cytoplasm through the Exportin-5 (Lund et al., 2004). Pre-miRNAs are finally cleaved within the cytoplasm by DICER RNase III, to generate double-strand mature miRNA. This causes the RISC (RNA-induced silencing complex) protein complex to selectively bind to the least thermodynamically stable single strand of the miRNA, while the unprotected single-stranded is rapidly degraded [albeit it can be detected to some extent in mouse and human cells (Schwarz et al., 2003)].

miRNA expression is profoundly altered in cancer, and miRNA may participate to carcinogenesis (Calin and Croce, 2006; Hamilton et al., 2013; Lin and Gregory, 2015; Pichler and Calin, 2015). Considering that single miRNAs can regulate 100 of genes, frequently in the context of a cell-specific network, targeting miRNA may prove effective to simultaneously modulate many different oncogenic pathways. Several important reviews have described the many roles and functions of miRNAs during oncogenesis, and have concluded that miRNA expression can serve as a reliable biomarker for patient with cancer (Tsongalis et al., 2013; Aquino-Jarquin, 2017; Rupaimoole and Slack, 2017; Subramaniam et al., 2019; Wang et al., 2019). For the later, miRNAs are involved very early during carcinogenesis and their level of expression reflects the patient's tumor stage (Lu et al., 2005; Balaguer et al., 2010; Hanoun et al., 2010). In addition, we (Buscail et al., 2015; Humeau et al., 2015) and others (Mitchell et al., 2008; Kosaka et al., 2010; Ono et al., 2015) found that miRNAs can be detected in body fluids such as saliva, serum, plasma and urine, paving the way for non-invasive molecular investigations (so called liquid biopsies). While Circulating Tumor Cells (CTCs), cell-free circulating tumor DNA (ctDNA), circulating tumor extracellular vesicles such as exosomes and blood-based protein markers are also promising blood-based circulating biomarkers, as extensively discussed elsewhere (Buscail et al., 2019); in this review, we intend to overview the interest of miRNA as a source of biomarkers for cancer diagnosis and prognosis. We will discuss the pre-analytical steps preceding miRNA quantification, and then review the most recent devices derived from micro- and nano-technologies for microRNA quantification and benchmark their performances with current standards.

## From Sample Preparation to miRNA Detection: Current Standards TOWARD Clinical Application

**Figure 1:**
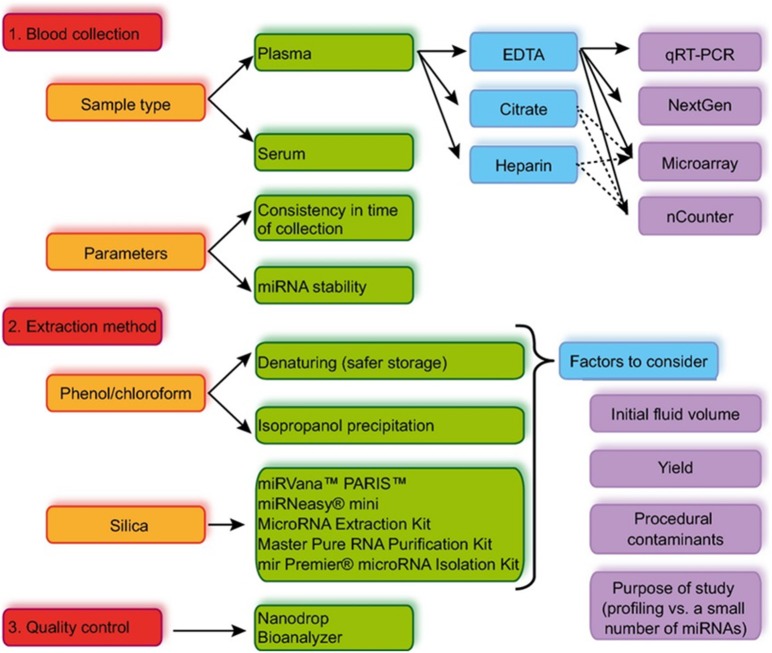
Summary of the standard work flow from sample collection to miRNA detection. Reproduced with permission from Moldovan et al. (2014).

Poorly controlled sample extraction can represent a source of many errors during detection (Figure 1). It is therefore essential to standardize protocols, to compare results and ensure trackability and reproducibility from one lab to the other (Faraldi et al., 2018, 2019). The current lack of consensus is one of the reasons that jeopardizes the widespread use of miRNAs as biomarkers. In addition to patient-related factors, such as age, exercise, diet, region of origin or medication, multiple steps in sample preparation can affect extracted miRNA amounts (Becker and Lockwood, 2013). In this section, we discuss the limitations and challenges associated with processing samples from biological fluids. Considering circulating miRNAs, we focus on the preparation of samples from whole blood products such as plasma and serum, as whole blood complexity exemplifies the main concerns found with other biological sources (Lawrie et al., 2008).

First, let's emphasize on the very high stability of miRNAs when protected by lipid or lipoprotein complexes, among others (El-Hefnawy et al., 2004). Studies showed that room temperature storage for 24 h has a minimal effect on the stability of endogenous miRNAs in human plasma (Mitchell et al., 2008). Storage at −80°C preserves miRNA content up to 10 months after extraction (Mraz et al., 2009). Similarly, storage for 2–4 years at −20°C very slightly affects miRNAs integrity, which is not the case for longer period of conservation (Grasedieck et al., 2012). Many studies have shown the presence of miRNAs in macrovesicles and exosomes (Gallo et al., 2012; Cheng et al., 2014; Joshi et al., 2015). In this way, exosomes isolation and concentration has been included in miRNA purification workflow to improve miRNA recovery rates. Some research groups even consider exosomes as potential cancer biomarkers (Properzi et al., 2013; Buscail et al., 2019), but it is now more widely accepted that miRNAs resistance to degradation relies to its binding to the Ago2 protein, that prevents from interaction with RNases in solution (Arroyo et al., 2011; Turchinovich et al., 2011).

The first step in pre-analytical operations is sample collection. The sampling tube influences the final quantity of miRNA collected, not only due to rapid degradation of RNAs in untreated tubes, but also because of adsorption to plastic. Kim et al. compared anticoagulant-free tubes, or tubes containing EDTA, heparin, sodium citrate, sodium fluoride or potassium oxalate (NaF/KOx). They next quantified miR-16 and miR-223 miRNAs using reverse quantitative transcription polymerase chain reaction (RT-qPCR) (Kim et al., 2012a). They found that using NaF/KOx treated tubes improves the yield of miRNA extraction by 2-fold when treating plasma, and by three-fold when using serum. Particular attention should be paid to hemolysis, as red blood cell lysis may pollute the final readouts for circulating miRNA analysis (Pritchard et al., 2012). To this end, a panel of miRNA (including miR-16, miR-92a, miR-451, and miR-486) is routinely used to qualify samples purity (Pizzamiglio et al., 2017).

Following sample collection, it is essential to isolate miRNAs from other components present in body fluids. These extraction steps are barely standardized and are principally based on phase separation extraction and/or silica columns that specifically adsorb RNAs. Such purification constraints are essential for proper enzymatic reactions during late-step detection. Phenol-chloroform extraction was first implemented in 1987 by Chomczynski and Sacchi (Chomczynski and Sacchi, 1987). This method associates phenol (C_6_*H*_5_*O*_5_) and chloroform (CHCl_3_) with a chaotropic agent, such as guanidinium thiocyanate ([CH_6_*N*_3_] + *SCN*^−^), to break down the 3D structures of proteins (more particularly RNases and RBPs, for RNA binding protein). This method separates proteins, DNA and RNA in a single step, and is compatible with small RNA sequences purification. Total RNA molecules from the aqueous phase are precipitated using isopropanol (or ethanol). However, recovering low-concentration RNA pellet (typically < 10 μg/mL) can be problematic, as the pellet can be difficult to visualize and therefore difficult to handle (Rio et al., 2010). Several groups reported that extending precipitation times coupled with centrifugations [20,000-xg for 1 h at 4°C (Hunter et al., 2008)], or glycogen addition improves recovery rates (Duy et al., 2015). Interestingly, phenol-based extraction efficacy varies with the sequence of the miRNA of interest (Kim et al., 2012b) leading to paper retraction (Kim et al., 2011). Silica column filtration to adsorb miRNAs by electrostatic interactions offers a frequent alternative to phenol-based extraction. Following filtration, rinsing steps are required to purify the sample with high efficacy. During this step, alcohol concentration is critical and must be carefully monitored for maximal miRNA recovery. Comparing such commercial kits efficiency is a first step toward standardization (Doleshal et al., 2008; McAlexander et al., 2013).

Quantification and qualification of RNA samples are crucial to address the purity and the integrity of the sample, as this will have direct consequences on detection (Fleige and Pfaffl, 2006), but also to illustrate the size profile and the RNA concentration within the sample of interest. For instance, degradation of the longest RNA fragments can lead to overestimation of miRNA levels (Kirchner et al., 2014). To date, spectrophotometry is the preferred method to quantify RNA with a sensitivity within the ng/μL range. However, this approach hardly distinguishes DNA from RNA in a complex solution. An alternative is to label nucleic acids with fluorescent dyes that show some degree of specificity with single or double-stranded DNA, RNA, or even miRNA (El-Khoury et al., 2016). These fluorescent markers interact electrostatically with nucleic acid chains and emit only when linked to a sequence. This strategy does not only enhance the selectivity but also increases the sensitivity of the approach (100 ng/mL range, Channavajjhala et al., 2014; Ge et al., 2014; Seashols-Williams et al., 2016). However, information on sample purity and size are still lacking. The later concern could be solved using electrophoresis, as this technique has the potential to separate and to quantify nucleic acids by size. This historical approach has recently been brought up to date by considering small size capillary or microfluidic chip, as the reduction of dimensions improves thermal dissipation, and therefore the application of larger electric fields, pushing the detection limits to tenth of ng/mL. Several technologies, such as Fragment Analyzer (AATI) in capillary electrophoresis or Bioanalyzer (Agilent) and the microLAS (μLAS) platform (Ranchon et al., 2016) in microfluidic chip format, are now routinely used in biological laboratories for sample qualification (Malbec et al., 2018).

MiRNA quantification methods have evolved from single molecule sequence analysis in days (Northern blot) to relatively short time-to-result quantitative RT-PCR and next-generation sequencing (NGS). However, there are critical challenges associated with small RNA sequencing library preparation, such as biased adapter ligation, formation of adapter dimers, the requirement to size-select the small RNA species, and the necessity to adapt for very low input protocols, especially if circulating biopsies are envisioned (Coenen-Stass et al., 2018). Additionally, direct quantification of specific miRNA sequences in samples requires post-analytical normalization protocols that are not yet standardized. While spiked synthetic miRNA are most often used, Faraldi et al. have recently identified hsa-miR-320d as a endogenous calibrator for RT-qPCR analysis (Faraldi et al., 2019). With the most advanced version of RT-PCR (RT-digital droplet PCR, ddPCR), amplification of a single sequence is performed in previously isolated “ready-to-use” microscopic drops (from nL to pL in volume) using microfluidic systems. Following the Poisson law, each drop contains 4–5 target molecules, strongly limiting the background noise. While more expensive than PCR, ddPCR is solely limited by the number of drops that can be generated. However, the detection of miRNAs using this technique requires implementing controls and optimizing enzymes, primers and probes, as demonstrated by Stein et al. (2017). In a similar way, miRNA quantification can be done using RNA or DNA microarray panels, with the recent emergence of Nanostring technology, a liquid hybridization approach with improved specificity compatible with a variety of sample types including cell lysates, exosomes and biofluids, that is to date routinely used in clinics (Wyman et al., 2011; Kolbert et al., 2013). However, this field of research is still dominated by direct sequencing, and more precisely next-generation sequencing (NGS). While the quantity of material collected following liquid biopsy might be limiting, NGS is the unique approach that holds the promise not only to detect genes, mutations or, more generally, specific sequences, but also to discover new sequences, nucleotide by nucleotide, including for miRNA research (Blondal et al., 2017).

To conclude, the cascade of operations leading to the quantification of miRNAs in biofluids can be cumbersome, time-consuming and crippled by serious flaws at all stages of the process, endangering proper quantification of the target analyte (Figure 1). Over the past 10 years, we have witnessed the emergence of new interdisciplinary programs for the titration of miRNAs, at the interface between biology and micro and nanotechnologies, propelled by the phenomenal progress of silicon technology for micro and nanoelectronics, aiming to provide new tools. The ultimate goal of these projects is to translate technological developments into clinical application for patient management.

## Micro and Nanotechnologies For the Detection of miRNA

Advances in the field of silicon micro and nanofabrication favored the reduction in size of electronic devices, notably dedicated to computing science, but also within the emerging field of sensors and actuators, with the promise to integrate various functions on a single chip. The first step toward biological applications was to generate devices for measuring chemical species concentration in solution. Biosensors were then referred to as any instrument that provides some sort of measurement in a biological system (pH, temperature, etc.) (Palchetti and Mascini, 2010). However, this notion evolved very quickly, as Clark defined in the early 1960s a biosensor as the combination of a biological recognition layer with a physical sensor, in that case electrodes coated with enzymes to measure oxygen concentration (Clark et al., 1958; Clark and Lyons, 1962). While measurements were at first mainly carried out by electrochemical sensors, the use of other transduction mechanisms such as optical, mechanical and electrical sensors was quickly implemented. In addition, biorecognition layers with improved specificity, *e.g*., antibodies, nucleic acids or synthetic ligands, were also developed.

Since the 1980s, microfabrication methods also fostered the emergence of microfluidics (Tabeling and Chen, 2005), defined as the production of fluid channels with dimensions between 1 and 100 μm (Whitesides, 2006). This strategy ensures fluid analysis in reduced volumes (nL-fL) with new opportunities for integration and automation. It is therefore perfectly understandable that reducing sensors size, as well as systems to manipulate biological objects, facilitates the investigation of rare molecular events (Tegenfeldt et al., 2004). In addition, the high-level of integration can couple several functions on the same analytical system. Such “laboratory on a chip” holds the promise of downscaling multiple functions of an analytical laboratory, following a “sample-in, answer-out” approach (Easley et al., 2006) that requires a wide variety of expertise in microfabrication, chemistry, biology, microfluidics and bioinformatics (Sahoo et al., 2007).

Coming back to miRNAs analysis, the first step toward micro and nanosensor-based quantification resides in the capture of selected targets by probe molecules and the production of measurable physical signals. Transducing biological recognition into a physical signal by means of micro- and nanofabricated sensors can take different forms (electrical, electrochemical, mechanical or optical) with specific foreseen advantages, such as the high-portability and multiplexing capability coupled to real-time and shorter analysis time. Because of large-scale fabrication capability of the microelectronics industry, these devices can generally be low-cost (although if produced in large quantities). However, limitations are also present: the reproducibility in terms of microfabrication and integration can be difficult to achieve, and, as discussed by Dahlin, reduced size does not always translate into better sensitivity [(Dahlin, 2012), Figure 2 and Table 1].

**Figure 2:**
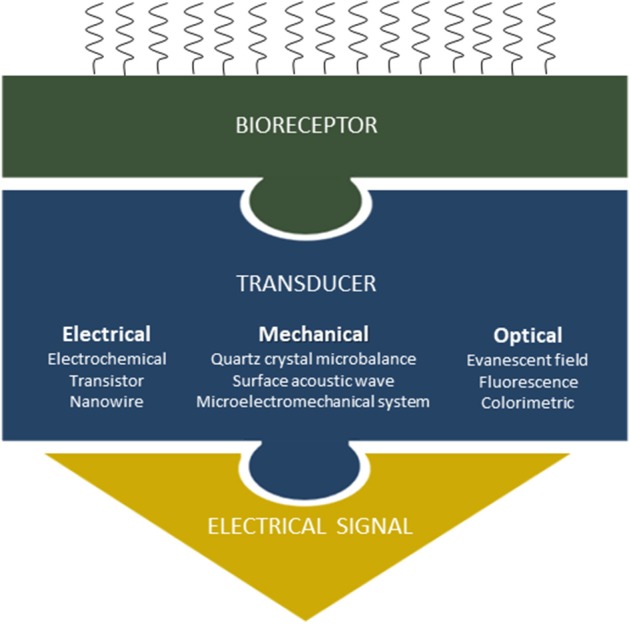
Transduction scheme principles: when the target miRNAs are captured by the probe molecules immobilized onto the sensor surface, a physical change can be measured through different transducing schemes.

**Table 1:** Main characteristics of miRNA detection using tools derived from micro- and nano-technologies.

**References**	**Approach**	**Device fabrication complexity**	**LOD**	**Sample complexity compatibility**	**Time to result** **(Real-time)**
Zhang et al. (2009)	Electrical	High	1 fM	Medium	1 h (no)
Tian et al. (2013)		Medium	50 pM	Low	< 10 min (yes)
Taller et al. (2015)		High	13 pM	Low	1.5 h (no)
Gao and Peng (2011)	Electrochemical	Medium	6 fM	Low	4 h (no)
Yin et al. (2012)		Medium	60 fM	Medium	5 h (no)
Dong et al. (2012)		Medium	67 fM	Low	1 h (no)
Tavallaie et al. (2018)		Medium	10 aM	High	2 h (no)
Johnson and Mutharasan (2012)	Mechanical	High	10 fM	High	~30 min (yes)
Duffy et al. (2018)		High	1 pM	High	~30 min (yes)
Šípová et al. (2010)	Optical	High	2 pM	Medium	<30 min (yes)
Qavi et al. (2011)		Medium	10 pM	Low	40 min (yes)
Degliangeli et al. (2014)		Low	5 pM	Medium	2 h (yes)
Joshi et al. (2015)		High	32.6 aM	High	12 h (no)
Liu et al. (2017)		Medium	0.6 fM	Medium	3 h (no)

*LOD, limit of detection. In the sample complexity compatibility column: “Low” signifies highly pure sample, “medium” if a sample preparation is necessary but not considered as limiting the detection time and “high” means that untreated samples can be detect. As a reference, the typical limit of detection of RT-PCR is 10 aM (unpublished results)*.

### Electrochemical Detection

Electrochemical detection involves oxidation/reduction reactions on a set of electrodes. Yin et al. performed electrochemical detection based on the use of gold dendritic nanoparticles functionalized with complementary, molecular beacon type probes targeting candidate miRNAs. These molecular beacons display a stable secondary structure, and they are engrafted on a graphene surface (*i.e*., graphite monolayer corresponding to crystalline hexagonally organized carbon atoms) that serves as a bonding layer. At the basal state, molecular beacons are found in a closed loop shape and do not emit any signal; however, the hairpin opens in the presence of target molecules after incubation during 2 h 30 at room temperature. Next, gold nanoparticles containing complementary strands of LNA (locked nucleic acid) are incubated for 2 h to interact with the end of the probe sequence. These nanoparticles also contain biotinylated DNA strands in order to interact with streptavidin molecules coupled to horseradish peroxidases for subsequent electrochemical amplification of the signal. Hydroquinone and hydrogen peroxide are then added to generate a current catalyzed by peroxidases, to indirectly indicate the number of strands hybridized with target miRNAs (Yin et al., 2012). Alternatively, OsO_2_ (Gao and Yang, 2006), RuO_2_ (Peng and Gao, 2011) or even silver clusters can be used (Dong et al., 2012). More recently, Travallaie et al. demonstrated miRNA detection directly into unprocessed blood through magnetic gold nanoparticles capture approach and collection onto a surface prior to electrochemical detection with a Limit Of Detection (LOD) down to 10 aM (Tavallaie et al., 2018). These detection methods benefit from relatively good sensitivities, but they require a multitude of steps and specific revelation chemicals that turn into complicated protocols and prevent from real-time detection.

### Electrical Detection

**Figure 3:**
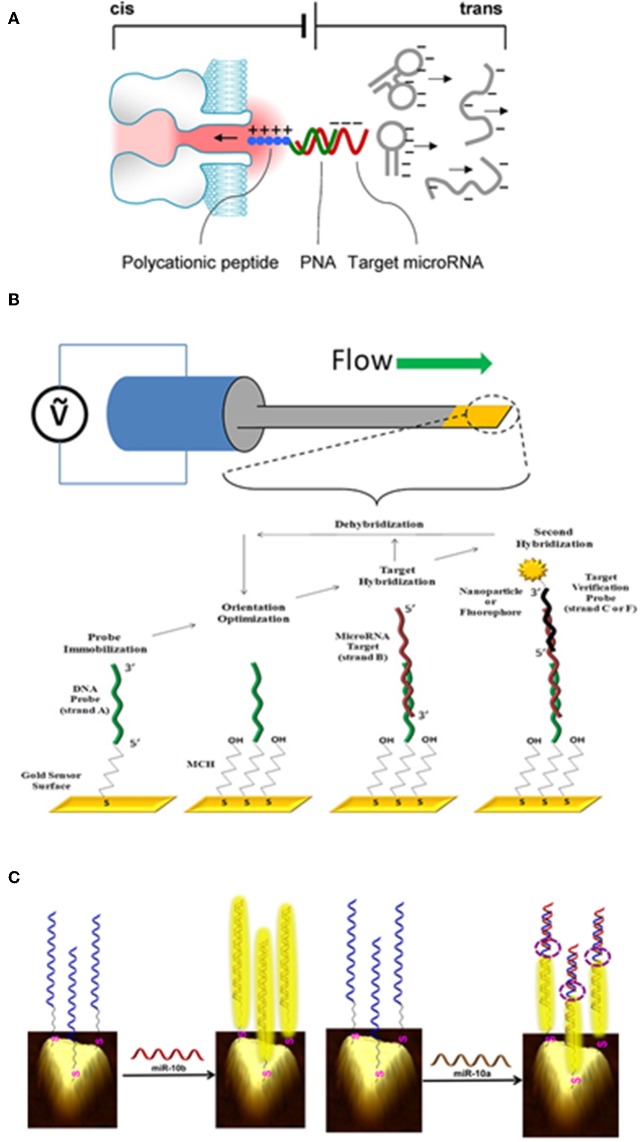
Example of miRNA detection using various micro- and nanofabricated biosensors. **(A)** Electrical detection of miRNA when passing through a nanoporous membrane. When applying an electric field through the membrane, target miRNAs that are hybridized to specific probes are drawn into the nanopore, while free nucleic acids move away from the pore. Reproduced with permission from Tian et al. (2013). **(B)** Mechanical detection of miRNA through a biofunctionalised cantilever. By applying a potential difference to the cantilever, its resonant frequency is measured. If a target miRNA interacts with the surface, a shift in the resonance frequency directly proportional to the added mass can be measured. Reproduced with permission from Johnson and Mutharasan (2012). **(C)** Optical detection of miRNA by surface plasmon resonance using gold nanoprisms. Reproduced with permission from Joshi et al. (2015).

Electrical detection of miRNAs is based on the measurement of a current or a potential difference following, for example, a change in the electrical resistance of a system after hybridization of the miRNA targets with dedicated probe molecules. It generally provides very low LOD at low cost. Nano-pores, which electrical resistance is modulated by the passage of RNAs, can be used in such setting. For instance, in the work of Tian et al., detection is carried out in two stages. First, selected miRNAs are hybridized with miRNA-specific PNA (Peptid Nucleic Acid) probes that are electrically neutral due to the lack of phosphate group in the nucleotide design. Each probe is also conjugated to a polycationic peptide (*i.e*., a positively charged peptide). The dipole formed upon hybridization with the miRNA target is then directed to the nanopore (formed within a lipid bilayer) via a strong electric field gradient. Unbound miRNAs remain negatively charged and move away from the nanopores due to the electric field. When miRNA/PNA complexes cross the nanopore, a current shift of a few picoamperes can be measured. This method is highly selective (detection of a single nucleotide difference), with a detection limit in the picomolar range. However, only proof of concept studies are performed to date, using synthetic miRNAs diluted in potassium chloride buffers (Tian et al., 2013). This suggests the presence of strong constraints associated with the use of a buffer solution that must be compatible with an electrical measurement and therefore does not allow for the processing of samples with minimal purification. Along the same line, PNA probes can be grafted onto silicon nanowires, using the amine group of the probe and the silicon surface modified with aldehydes. Because the target miRNA is negatively charged and the PNA probe is electrically neutral, the accumulation of electrical charges modifies the resistance of the nanowire that is translated into miRNA content. This method offers a very good specificity with single base pair discrimination and detection level in the femtomolar range (Zhang et al., 2009). However, manufacturing these devices remains challenging, and incubation and detection must be performed in low ionic strength buffers. A similar strategy can be applied to ion-exchange nanomembranes, where the current voltage characteristic across the membrane is directly correlated to the hybridization rate due to miRNA accumulation. The use of ion-exchange membranes was applied to the detection of miRNAs in cell extracts from pancreatic cancer origin, along with an integrated module for lysing exosomes-containing miRNAs using surface acoustic waves (Figure 3) (Taller et al., 2015).

### Mechanical Detection

Mechanical changes induced by miRNA hybridization on miniaturized suspended structures can be detected and serves for mRNA quantification. This technology, called MEMS for microelectromechanical systems, can be used in static or dynamic modes. In the first case, the capture of target molecules at the surface of the structure results in additional mass and stress, causing a deflection that can be quantified by means of various transduction mechanisms (mainly optical) (Duffy et al., 2018). Alternatively, these systems can be used in dynamic regimes, *i.e*., in vibration mode, where a decrease in the structure resonance frequency is due to the addition of mass on the surface of the resonator. The dissipation (damping) constant, corresponding to the viscoelastic properties of the material bound to the membrane, may give additional information, notably on the nature of the interactions and the captured molecules organization. Few studies have reported the use of MEMS for miRNA detection. Beams made of silicon zirconate, a piezoelectric material deformed by the application of an electric field, with a surface area of about 1 mm^2^ covered with a 100 nm thick layer of gold grafted with thiolated DNA probes complementary to a miRNA target, are one of the most promising MEMS devices for miRNA quantification, as shown by Johnson et al. (Johnson and Mutharasan, 2012). The shift in resonance frequency due to miRNA:probe interaction is directly proportional to the added mass. The measurement is made via periodic electric field (100 mV) application for mechanical excitation of the beam (Figure 3). This technique offers a good selectivity and is label-free. It is also compatible with detection in complex fluids such as serum. However, reducing the dimensions of MEMS to improve its sensitivity is still a challenge that remains unsolved as well as minimizing the energy loss due to the operation in liquid medium that leads to reduced quality factors, hence performances.

### Optical Detection

Optical methods that have a long tradition in biosensing can also be very helpful for the quantification of miRNAs. Our group recently demonstrated that spatially resolved nanofluidic-embedded biosensors supported by fluorescence microscopy authorizes real-time, fast and direct discrimination of single-nucleotide difference (SND) within oligonucleotide sequences in a single step interaction (Cacheux et al., 2018). For this, we designed sensors with much larger linear dimension as compared to the channel depth, with the objective to limit the interaction by the convection rate over the whole sensor. Consequently, targets are fully collected, inducing a non-uniform spatial hybridization profile over the sensor. We managed to discriminate single base pair mismatches on miRNAs sequences by optimizing the interaction temperature and the probe design. This strategy could be applied to any surface-based biosensing transduction scheme, *e.g*., surface plasmon resonance (SPR) imaging, assuming the integration of nanofluidic channels. Along this line, Šípová et al. used thiolated DNA probes grafted on a gold surface for miRNAs bio-recognition (Šípová et al., 2010). Following hybridization of the probe with the target miRNA, monoclonal antibodies specific of DNA/miRNA interactions are added to amplify the change in refractive index leading to a detection range in the picomolar level after 30 min of interaction. Magnetic beads functionalized with specific probes coupled to the trombusvirus-encoded suppressor of RNA p19 further improves the sensitivity by capping the miRNA/probe duplex to reach the femtomolar range (Nasheri et al., 2011). Fang et al. designed a very similar detection method, while more complex but with similar sensitivity. In their setting, LNA probes are captured on the surface. Then, they perform poly(A) tail reaction following hybridization of the miRNA with the probe. Gold nanoparticles containing poly(T) strands hybridize with the newly constituted miRNA poly(A) tail, and detection is performed using SPR, as gold nanoparticles amplify the signal (Fang et al., 2006). Similarly, the work of Liu et al. also supports the use of SPR for the detection of miRNAs. In their work, nanoparticles are modified with aptamers, resulting in very high selectivity. Detection is performed in complex media such as diluted serum, after 50 min of interaction and several stages of revelation, to reach excellent detection levels (~100 fM) (Liu et al., 2017). Last but not least, Korc's team recently obtained very impressive results using gold nanoprisms for the detection of miRNA sequences in biological fluid extracts as well as after isolation of circulating exosomes (Joshi et al., 2015) (Figure 3). Detection limits reported are very low, in the order of 100 attomolar with excellent selectivity. However, important errors can be noted when detecting high concentrations of miRNAs (10 pM −10 nM). In addition, time-to-result is very long (~12 h) and detection cannot be performed in real time. Another approach, very similar to SPR, calls for silicon micro-rings resonators. DNA probes are linked to silicon via silane functions and hybridized with target miRNAs. Adding S9.6 antibodies specific of the miRNA/DNA interaction amplifies the detection signal, following optical excitation using an external laser. The protocol used in this work is much simpler (Qavi et al., 2011), but the detection threshold is relatively high compared to other optical methods, albeit probe design increases sensitivity (Graybill et al., 2016).

Fluorescence measurements based on fluorophores or quantum dots, which emit light at a specific frequency, have recently emerged as reliable technological approaches to quantify miRNAs. These, detection methods generally involve labeling steps. In their work, Degliangeli et al. immobilized thiolated probes specific to miRNAs on a layer of gold nanoparticles. The surface is then passivated with PEG (Polyethylene Glycol) to limit non-specific interactions. Following DNA probe hybridization with miRNA targets, DNA/miRNA duplexes hydrolysis using endonucleases provokes the release of the fluorophores engrafted on the probes and initially quenched by the gold nanoparticles, and emission of a fluorescent signal that can be measured with a limit of detection of 5 pM (Degliangeli et al., 2014). Due to their excellent optical properties (control of excitation and emission wavelengths, minimal photobleaching, etc.), quantum dots recently became serious alternatives for miRNA detection. Su et al. generated miRNA-specific DNA probe associated with a quencher on one side, and with a quantum dot on the other side. In the presence of miRNA, the quantum dot fluorescence signal is quenched, due to absorption by the organic quencher (BHQ2) from the DNA probe, and fluorescence measurement can reveal fM of target sequences (Su et al., 2014). Another alternative is the use of 2D graphene structures that adsorb and quench the fluorescent of single-stranded sequences specific to target miRNA. When the miRNA target interacts with the surface, duplex is formed, and desorb. Thus, the fluorophore labeled probes move away from the graphene surface, and fluorescence can be measured (Zhu et al., 2015). Other research groups have coupled this approach with more elaborated probe designs such as UNA (unlocked nucleic acid) bases with improved selectivity (Robertson et al., 2017). Along these lines, lateral flow immunoassay analysis (LFIA), also called lateral flow nucleic acid biosensor (LFNAB), can be preferred for microRNA quantification. In LFNAB, DNA-gold nanoparticles based lateral flow nucleic acid biosensor are used for visual detection of microRNA in aqueous solutions and biological samples with low-cost and short analysis time (Gao et al., 2014). Interestingly, the assay can be multiplexed (Zheng et al., 2018). In the later study, such biosensor could successfully detect multiple microRNAs in spiked serum samples without cross-reactivity and matrix-effect. LFNAB detected candidate miRNAs with LOD ranging from 0.061 to 0.085 nM, that may open for complementary clinical application, particularly when resources are limited.

## What are the Next Steps to Convert miRNA Detection Using Micro- and Nano-technologies Into Clinical Application?

As stated before, miRNAs are key players in cancer and can now be considered as a reliable source of biomarkers. They are highly stable and can be detected in body fluids, with the promise to ensure patient stratification. In some specific cases, miRNA detection may be more effective in detecting smaller tumors, micrometastases or early relapse following surgery, notably in pancreatic cancer patients (Buscail et al., 2019). However, miRNAs are not yet routinely used for cancer management. Besides the validation of proper miRNA signatures indicative of cancer by biologists and clinicians, one reason for this could be the lack of robust, reliable, portable, low-cost, specific, and sensitive technologies at the bed side to ensure miRNA quantification in routine practice.

Several technological barriers must be overcome for detecting miRNAs directly in complex fluids (plasma, saliva, urine, etc.). First, circulating miRNAs are most of the time trapped with proteins or within exosomes in biofluids. It is thus necessary to devise specific technological bricks to release these low molecular weight RNA molecules before proceeding to analysis and detection. Interestingly, Taller et al. created a device with the ability to lyse exosomes-containing miRNA directly on a chip (Taller et al., 2015). Unfortunately, the electrical detection system is not compatible with complex fluids, strengthening the fact that the whole picture (pre-analytic and analytic steps, specific targets or target environments) must be considered to generate the best technological offer.

The next key step is to better address sample preparation. For biologists, standard amplification-based detection methods require high levels of purity due to the critical sensitivity of enzymatic reactions. Generally, extraction protocols favor quality rather than quantity, which may represent a major issue when dealing with devices that require relatively high amounts of starting material. One way to achieve this goal would be to invent a novel microfluidic technology for the concentration and separation of oligonucleotide sequences. This is already true for DNA fragments, using the lab-on-chip system μLAS developed by Bancaud et al. that concentrates, separates, and detects nucleic acids in a single module (Malbec et al., 2019). Target concentration is achieved by applying an electric field opposite to the solution flow of viscoelastic liquids. It would be interesting to develop this technology for the concentration of miRNA sequences.

Several groups are actively working to increase the sensitivity of micro- and nano-technology derived platforms to improve miRNA detection in liquid biopsies. Their goal is to provide novel devices that detect and quantify several candidate biomarkers, ideally from different molecular classes, as composite signatures, such as Cancer-SEEK, that recently revealed their clinical value for cancer early detection and follow-up (Cohen et al., 2018). The coupling of several targets on the same detection platform remains a scientific challenge with strong clinical potential. One interesting option for multiple functionalization (with resolutions in the order of 10 μm) could be the use of microcontact printing or tools for drop delivery, derived from MEMS technology and capable in theory of sampling and depositing very small volumes of solution (Berthet-Duroure et al., 2008; Salomon et al., 2012). Such experiment would be a first step toward multiplexing detection systems.

This synergy of expertise may ultimately pave the way for implantable biosensors that can evaluate disease indicators, such as miRNAs, in real-time in human bodies. While biocompatibility and long-term stability are still being addressed, prototypes have recently emerged for the management of patients with various diseases. As an example, wireless sensor implant targeting real-time blood glucose body levels were recently developed by a molecular nanotechnology company expert in MEMS called Zyvex^1^. This novel portable device does not only indicate glucose levels but also communicates with the clinical management system. Extra-body circulation systems may represent a first step to address miRNA quantification in blood of patients with cancer, for the first time in real-time and longitudinal studies. Thus, by and large, micro and nanotechnologies offer significant promise in the medical device community. They may also pose several regulatory challenges, which as time goes by, will probably become more pressing than the technical challenges. Nevertheless, new technologies have the potential to revolutionize cancer management when associated with the right candidate biomarkers for the right patient or disease state, to help manage patients with serious diseases such as cancer, following a precision medicine strategy.

## Author Contributions

PC wrote a draft and finalized the manuscript. JC built Table 1 and inserted figures and the biobliographic references. JC, TL, and AB significantly edited the manuscript.

### Conflict of Interest

The authors declare that the research was conducted in the absence of any commercial or financial relationships that could be construed as a potential conflict of interest.

## Footnotes

^1^https://www.zyvexlabs.com/
